# Interactions among Zinc, Iron, and Paraquat in the Physiological and Toxicological Responses of the Egyptian Cotton Leafworm *Spodoptera littoralis*

**DOI:** 10.3390/toxics13010038

**Published:** 2025-01-05

**Authors:** Haq Abdul Shaik, David Siaussat, Archana Mishra

**Affiliations:** 1 Institute of Entomology, Biology Centre, CAS, Branišovská 31, 370 05 České Budějovice, Czech Republic; 2 Institut d’Ecologie et des Sciences de l’Environnement de Paris (iEES-Paris), Sorbonne Université, CNRS, INRAe, IRD, Université Paris Créteil, Université Paris cité, F-75005 Paris, France; david.siaussat@sorbonne-universite.fr; 3 IRRI South Asia Regional Center, Delhi 110054, India; archana.shaik@irri.org

**Keywords:** co-interaction, immunomodulation, pest management, physiological toxicity, pollutants, sublethal exposure

## Abstract

Agricultural pollutants co-interact and affect the vital functions, stress tolerance, resistance, immunity, and survival of insect pests. These metal–herbicide interactions have inevitable but remarkable effects on insects, which remain poorly understood. Here, we examined the effects of the interactions among zinc (Zn), iron (Fe), and paraquat (PQ) at a sublethal dose on the physiological response of the Egyptian cotton leafworm *Spodoptera littoralis*. Co-exposure to Zn and Fe improved leafworm survival (100% at 10–20 mg, 85% at 40 mg) compared to separate exposures. Low Zn/Fe/PQ toxicity likely stemmed from metal complexes having efficient chelating activity, enhancing resilience. Low exposure to Zn, Fe, and Zn/Fe increased food intake and larval weight and affected frass production. Interestingly, the combined application of Zn/Fe/PQ increased larval and pupal weight in surviving individuals. Zn/Fe was found to be crucial in the ecdysis of larvae into pupae, resulting in reduced larval mortality and a prolonged pupal ecdysis duration (% days). Providing important information regarding physiological responses and pest management, this study demonstrated the realistic conditions caused by the interactions of biological trace elements, such as Zn and Fe, with PQ. A disc diffusion susceptibility test in hemolymph bacteria revealed differences among Zn, Zn/Fe, and Zn/Fe/PQ, suggesting that their interaction might play an immunomodulatory role in *S. littoralis*.

## 1. Introduction

Agricultural pollutants such as metals and herbicides originate from farming and irrigation practices globally, including manure, fertilizers, pesticides, and industrial waste by-products [1]. Such pollutants negatively impact all trophic levels and, hence, the sustainability of natural ecosystems. Primarily, metal accumulator plants such as *Noccaea ochroleucum* that are exposed to pollutants absorb them from the soil through their roots, transport and accumulate them in their leaves, and ultimately metabolize and detoxify the excess [2]. Phytophagous insects are indirectly exposed to these pollutants when they attack and feed on these plants, impacting their life cycles [3,4]. Consequently, increasing numbers of studies are investigating the effects of various metals, including zinc (Zn) and iron (Fe). At very low concentrations, these metals are vital bio-elements, with beneficial effects, while, at higher concentrations, they are considered to be pollutants, with toxic and deleterious effects on insects’ vital functions, such as stress tolerance, resistance, and survival. As a response, insects develop resistance through behavioural changes (avoiding treated areas), physiological barriers (reduced absorption), and metabolic detoxification. They may also alter target sites to neutralize pollutant effects or exhibit cross-resistance to similar chemicals [5].

Furthermore, while herbicides are widely applied to crops, their unwanted effects on non-target organisms in agroecosystems are being increasingly questioned [6]. Paraquat (PQ) is one such example and is one of the most widely utilized global herbicides. However, it is important to understand how the combined effects of metal pollutants and herbicides at low or sublethal doses influence pest insects and their broader impact on the surrounding ecosystem. Studies indicate that PQ exposure impacts *Drosophila melanogaster* Meigen (Diptera: Drosophilidae) in various ways, including reducing longevity [7,8], reducing the number of dopaminergic neurons [9], changing dopamine and dopamine metabolite levels [9], and altering motor function [10].

Similarly, Yan et al. [11] examined the risk of heavy metal exposure in insect pests, its impact on pest prevalence, and its role in developing cross-tolerance to insecticides.

In fact, there is a growing literature base showing that these realistic conditions of co-exposure deleteriously influence insects through both inhibitory and stimulatory effects [12,13]. Insects provide excellent models to explore these questions, as there is already a wealth of information on the impact of single contaminants and pollutant mixtures on various biological processes, including growth, deformities, reproduction, gene expression, motor activity, and hormone levels [14,15,16,17,18,19]. Hormetic and endocrine disrupting effects are often observed at low concentrations of pollutants and have also been demonstrated across various insect species, especially in Diptera [20]. There is also a paucity of research on how pollutants impact Lepidoptera. One model insect is the lepidopteran pest Egyptian cotton leafworm *Spodoptera littoralis* Boisduval (Lepidoptera: Noctuidae), a polyphagous pest native to Africa and the Mediterranean region. Biologically, it has high reproductive potential and adaptability, making it a resilient species. Ecologically, it thrives in diverse habitats, feeding on over 100 plant species. Agriculturally, it is a major pest in crops like cotton, maize, and vegetables, causing significant economic losses globally. Categorized as a major pest species, it is increasingly being used to assess the effects of pollution, including toxicological and pest management, as well as its resistance against chemicals, pesticides, and biological agents [21,22]. The life cycle of *S. littoralis* lasts for around a month, allowing key life parameters to be easily recorded, including mortality, development, fecundity, and lifespan.

Several studies have highlighted the effects of zinc (Zn) on the *Spodoptera littoralis* genus. For instance, Sharaby et al. [23] demonstrated that ZnSO_4_ is toxic at a low concentration (25 mg/mL) and acts as a growth disruptor. Similarly, Ali et al. [24] reported that Zn (150 μg/g) modulates the fitness of *Spodoptera littoralis* larvae. Interestingly, Zn also exhibits a protective effect against cadmium (Cd) exposure, as shown by Tarnawska et al. [25], with this effect extending over multiple generations in *Spodoptera exigua* Hübner (Lepidoptera: Noctuidae). These studies collectively highlight Zn’s dual role as both a toxicant and a protective agent under dose-specific conditions.

In comparison, the toxicity of iron (Fe) in Lepidoptera species remains poorly studied. However, research on other insects has provided some insights. For example, Slobodian et al. [26] investigated Fe toxicity in *D. melanogaster* and found that exposure to 5 mM Fe during the embryonic period delays hatching in the F1 generation due to reduced cell viability in the ovarian tissue. In *Apis mellifera* Linnaeus (Hymenoptera: Apidae), He et al. [27] revealed that Fe overload caused by the insecticide imidacloprid induced oxidative stress, leading to significant mortality. Current research is still exploring the mechanisms linking pesticide toxicity and Fe metabolism, underscoring the need for further investigation. Fe and PQ act as synergistic environmental risk factors promoting age-related neurodegeneration [28]. FeSO_4_ at 0.5–20 mM concentrations reduced the survival and mobility of *D. melanogaster* compared to 20 mM PQ [10]. Furthermore, metal chelators might mitigate PQ neurotoxicity [29]. Thus, the impacts of Zn, Fe, and PQ on insect pests are clearly interconnected, impacting vital parameters. Yet, the research on the co-interactive effects of metal (Zn, Fe) and herbicide (PQ) toxicity remains limited. Thus, here, the interactions among Zn, Fe, and PQ in the physiological and toxicological responses of *S. littoralis* were examined due to their ecological relevance and susceptibility to these pollutants. The experimental design incorporated controlled exposures to simulate realistic environmental conditions, enabling a detailed assessment of physiological and toxicological impacts.

## 2. Materials and Methods

### 2.1. Spodoptera littoralis Rearing and Management

The larvae of the Egyptian cotton leafworm *S. littoralis* were reared with the relative humidity of 25 ± 1 °C and a 16:8 h light–dark photoperiod. They were fed with a semi-artificial diet prepared by using the protocol established by the insect physiology laboratory. Specifically, (1) 500 g small white beans was cooked until soft, excess water was removed, and the beans were homogenized in a blender; (2) 75 g dry yeast was cooked in 300 mL distilled water, to which 7.5 g ascorbic acid with a purity 99% (Sigma-Aldrich, Darmstadt, Germany), 1.25 g sorbic acid (Sigma-Aldrich, Darmstadt, Germany), and 4.75 g methylparaben (Sigma-Aldrich, Darmstadt, Germany) were added successively; and (3) a hot solution of 30 g agar in 500 mL water (Base diet) was prepared, and the desired concentration (10, 20, 40 mg/kg diet) of agents for a semiliquid diet was added and poured into Petri dishes (20 cm diameter). These concentrations were selected based on our previous results, which provided a rationale for their relevance and effectiveness in this context. After cooling to room temperature, the dishes were stored in a refrigerator at 4 °C, and the diet was used for one month. Larvae were kept in groups of about 15 individuals/treatments that were checked at 24 h intervals. Only newly ecdysed larval instars were transferred to separate plastic cups (40 mL volume) for use in the study. Each cup was lined with a 5 cm disc of Whatman filter paper (Sigma-Aldrich, Darmstadt, Germany). Around 10 g/day (after weighing) was provided to the larvae on a small piece of aluminum foil; this is referred to as a dose in this manuscript.

### 2.2. Source of Metals (Zn, Fe) and Herbicide (PQ)

The salts of Zn in the form of ZnSO_4_ and Fe in the form of FeSO_4_, along with paraquat (PQ), were purchased from commercial suppliers (Sigma-Aldrich, Darmstadt, Germany). All chemical reagents were of analytical grade and were used as received without further purification. First, 1M dilutions of all chemicals (Zn, Fe, and PQ) were prepared in water and added, with adequate stirring, to the semiliquid diet at the desired concentrations of 10, 20, and 40 mg/kg diet for the treatments. The water used in the preparations of all solutions was purified by deionization and filtration with IWA 30 iol WATEK apparatus (Ledeč nad Sázavou, Czech Republic) to a resistivity higher than 18.0 MΩ cm.

### 2.3. Toxicity Tests, Survival Pattern, and Single and Mixed Exposure of Metals (Zn, Fe) and PQ in S. littoralis

For LC20, diets containing various concentrations of Zn, Fe, and PQ were fed to 2nd larval instars of *S. littoralis* (15 larvae/diet/Petri plate). The toxicity of the chemicals was tested against the mortality (%). The resulting, LC20 level was applied to all treatments to represent a low-concentration context. To determine the toxicity and survival pattern, 2nd-instar larvae of a similar weight (about 120 mg) were collected and placed in 32 cm Petri dishes (15 larvae/diet/Petri plate). They were then exposed to different artificial diets containing metals (Zn, Fe, Zn/Fe), termed pre-PQ diets (before PQ application), and herbicides (PQ, Zn/PQ, Fe/PQ, Zn/Fe/PQ), termed as post-PQ diets (after PQ application). The controls were fed a base diet with distilled water. Each larva was monitored at 24 h intervals.

**Figure 1:**
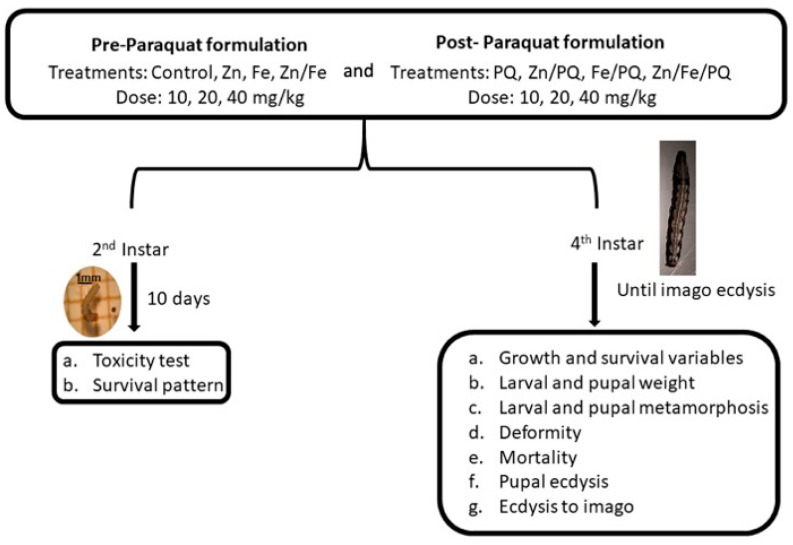
Schematic representation of *S. littoralis* pre- and post-PQ treatments, doses, larval stages, the duration of treatment, and study variables.

At the beginning of the 4th larval instar (about 180 mg), larvae were fed with artificial diets containing pre- and post-PQ combinations. Specifically, we monitored growth and survival variables, larval and pupal weight, larval and pupal metamorphosis, deformity, mortality, pupal ecdysis, and ecdysis to imago (Figure 1). Larval weight (mg), diet consumption (mg), and frass (mg) were measured until the control larvae began pupating, which took approximately 48 h. The experiment was repeated three times (15 larvae/groups) for the statistical analysis.

### 2.4. Interactions Between Metals (Zn, Fe) and Herbicide (PQ) and Their Impact on Antimicrobial Activity

The pro leg of the larva was cut off, and the hemolymph was streaked on the nutrient agar (NA) plates containing 0.8% Difco Bacto nutrient broth and 1.2.% agar [30]. Plates were incubated at 25 °C. Characteristic colonies of bacteria developed after 1–2 days were sub-cultured 2–3 times until a homogenous culture was obtained. The culture was transferred to nutrient agar slants, incubated at 25 °C for 3D, stored at 6 °C, and sub-cultured at least once every month. Using the disc diffusion assay, we checked the sensitivity of *S. littoralis* hemolymph bacteria by applying single- and mixed-metal herbicide formulations (Zn, Fe, Zn/Fe, PQ, Zn/PQ, Fe/PQ, and Zn/Fe/PQ) to streptomycin antibiotics. Ten circles of filter paper of 0.5 cm diameter were moistened with 30 µL water containing 30 µg streptomycin and 30 µg of pre- or post-PQ treatment doses, respectively. Small Petri dishes (diameter of 3.5 cm) were employed with a single disc in each Petri dish, and the treatments were applied separately to avoid cross-contamination. The plates were incubated at 30 °C for 1–2 days to check the appearance of the zone of inhibition and for further quantification. Each treatment included 5 specimens, meaning that in total, 40 larvae were tested.

### 2.5. Statistical Analysis

The lethal concentration (LC_20_) values of Zn, Fe, and PQ were calculated manually using Probit analysis at 95% confidence intervals. Kaplan–Meier survival analysis was conducted to test the proportion of survival and median survival time after pre- and post-PQ treatments. Survival curves were compared using the log-rank test. A comparison of groups to find statistical differences was performed via two-way ANOVA at a significance level of *p* < 0.05. Tests of the homogeneity of variances confirmed the normal distribution of the dataset. *F*-tests included degrees of freedom and the degrees of freedom of the error (within-group degrees of freedom), and the means were separated using the Bonferroni multiple comparison test. For the antibiotic effect, one-way ANOVA and Dunnett’s multiple comparisons tests, *p* < 0.05, were performed thrice. Different letters indicate significant differences in the treatment. Points in the bar graphs represent mean ± SEM. For all statistical analyses, GraphPad Prism version 10.0.0 software (San Diego, CA, USA) was used.

## 3. Results

We uncovered new insights into the toxicological effects of metal (Zn, Fe) and herbicide (PQ) co-application on *S. littoralis*, taking respective LC20 ranges into account (Table 1). The lethal concentration (LC20) values of Zn, Fe, and PQ represent the concentration of the compound required to cause 20% mortality in the test population. The analysis result showed the following: Zn (slope = 5.474 + −1.235 chi-square = 5.0341, DF = 4); Fe (slope = 4.990 + −1.091, chi-square = 3.248, DF = 4); and PQ (slope = 4.108 + −0.832, chi-square = 5.8722, DF = 4). We ensured precise estimation by accounting for the dose–response relationship, providing statistically reliable data for evaluating the toxicity thresholds of these compounds under specified conditions (Table 1).

### 3.1. Survival Pattern and Synergistic and Antagonistic Action of Metals (Zn, Fe) and Herbicide (PQ)

**Figure 2:**
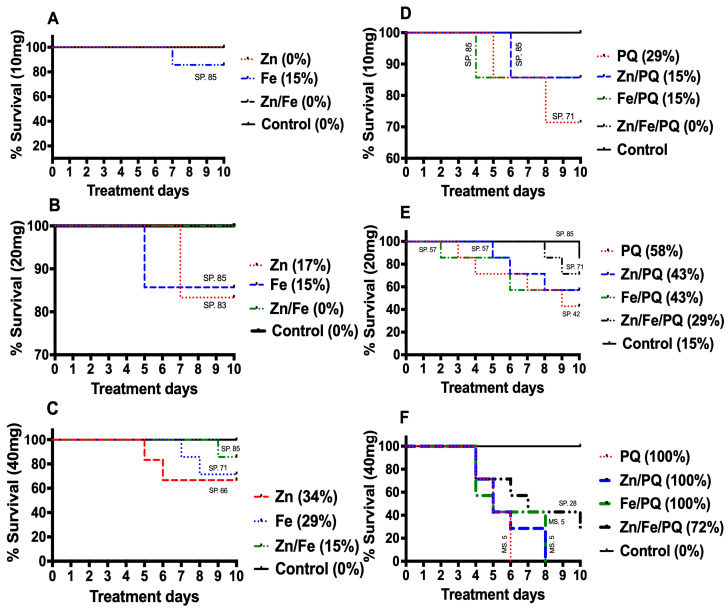
(**A**–**F**) Kaplan–Meier survival curves of *S. littoralis* larvae that were single and co-exposed to metals and herbicide in the newly ecdysed 2nd larval instar until the controls initiated pupation (10 days). Larvae were fed with a base diet (control), Pre-PQ (**A**–**C**), and Post-PQ (**D**–**F**). Each treatment included 15 larvae, with 360 in total being tested on. There were three replicates. SP (survival proportions in days) is shown on the left y-axis, and (%) mortality for treatments is shown on the right y-axis.

The survival patterns of larvae changed between those that were singly exposed and those co-exposed to Zn, Fe, and PQ. Survival rates followed similar curves but differed with respect to time of death and morphological abnormalities, showing that the cause of death was dose-, combination-, and compound-dependent (Figure 2A–F). Specifically, larval mortality began after 7 days with 10 mg Fe but began after 4, 4, and 8 days for PQ, Zn/PQ, and Fe/PQ, respectively. At 10 days, the total mortality was Fe (15%), PQ (29%), Zn/PQ (15%), and Fe/PQ (15%) (Figure 2A,D). No mortality was recorded in the control, Zn, Fe, or Zn/Fe/PQ treatments. For the 20 mg dose, the mortality at 5–7 days was control (0%), Zn (17%), Fe (15%), and PQ (58%); at 2–10 days, it was Zn/PQ (43%), Fe/PQ (43%), and Zn/PQ/PQ (29%) (Figure 2B,E). All treatments exhibited mortality at 5–9 days with a dose of 40 mg, except for Zn/Fe/PQ (72% mortality), while the total mortality at 10 days remained at 100% (Figure 2C,F). Zn/Fe clearly enhanced survival (10, 20 mg 100%; 40 mg, 85%), with the Zn/Fe/PQ complex lowering individual compound toxicity and prolonging the survival time of larvae (Figure 2A–F).

The survival of larvae in the 10 mg dose of Zn, Fe, Zn/Fe, PQ, Zn/PQ, Fe/PQ, and Zn/Fe/PQ was 83, 85, 100, 71, 85, and 100%, respectively. At 20 mg, it was 100, 85, 100, 42, 57, 57, and 71%, respectively. At 40 mg, it was 66, 71, 85, 0, 0, 0 and 29%, respectively. Compared to the control, Kaplan–Meier data showed that the survival rate differed for larvae exposed to 10 mg (log-rank 2 = 5.027; df = 7; *p* = 0.65), 20 mg (log-rank 2 = 13.73; df = 7; *p* = 0.05), and 40 mg (log-rank 2 = 44.8; df = 7; *p* = 0.001) (Figure 2).

### 3.2. Influence of Single and Co-Exposure to Zn, Fe, and PQ on Larval and Pupal Development

**Figure 3:**
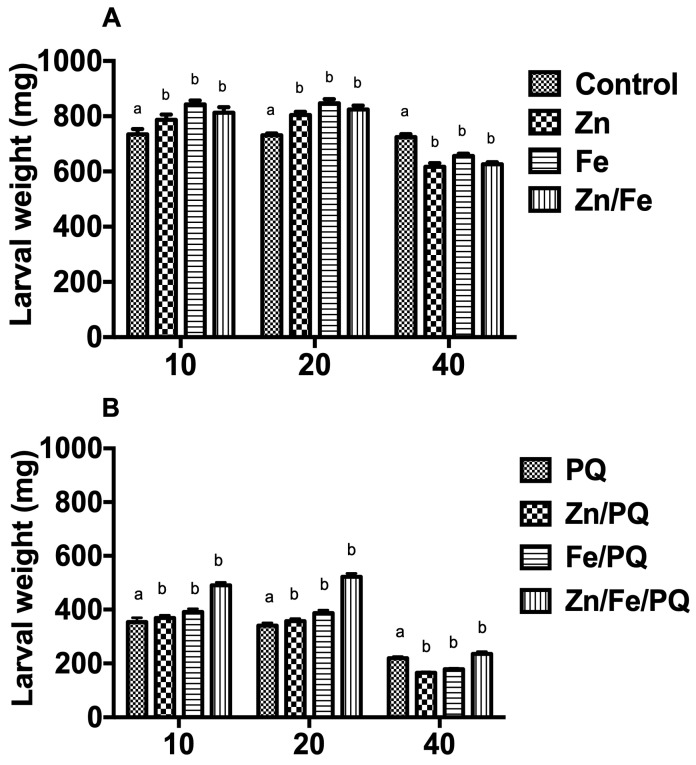
(**A**,**B**) Dose-dependent (10, 20, 40 mg/kg) influence of single exposure and co-exposure to Zn, Fe, and PQ on the larval weight of *S. littoralis*. Larval weight (mean values in mg per caterpillar) was recorded after 48 h for the different diet exposures (fourth larval instar). Each treatment included 15 larvae, with 360 being tested in total. The experiment was repeated three times. Points on the bar graphs represent mean ± SEM. Different letters indicate significant differences in the treatment groups with the respective controls (pre-PQ–controls; post-PQ–PQ) (two-way ANOVA, Bonferroni post hoc tests, *p* < 0.05).

**Figure 4:**
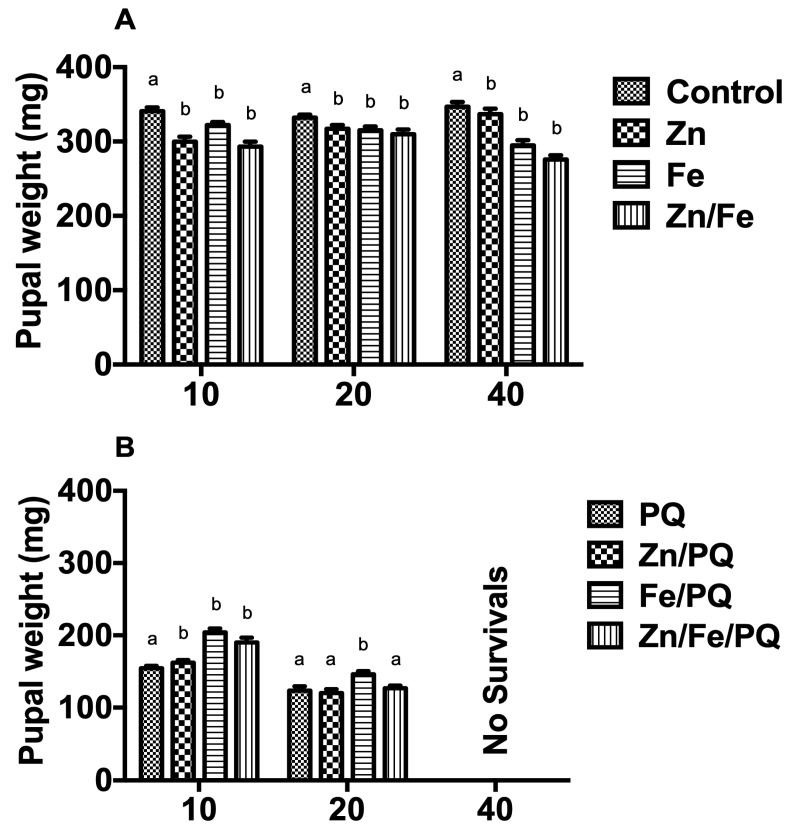
(**A**,**B**) Dose-dependent (10, 20, 40 mg/kg) effect of the single co-exposure of Zn, Fe, and PQ on the pupal weight of *S. littoralis*. Pupal weight (mean values in mg per pupae) was recorded for emerging pupae exposed to the treatments. Each treatment included 15 larvae, with 360 in total being tested. The experiment was repeated three times. Points on the bar graphs represent mean ± SEM. Different letters indicate significant differences in the treatment groups with the respective controls (pre-PQ: controls; post-PQ: PQ) (two-way ANOVA, Bonferroni post-tests, *p* < 0.05).

Single and mixed exposure to metals and herbicides affected larval (Figure 3A,B) and pupal weight (Figure 4A,B). The effects of single and mixed exposure to chemicals significantly differed, showing a dose-specific pattern (two-way ANOVA, *p* < 0.0001, F = 397.3; *p* < 0.0001, F = 396.3). Compared to the control, the body weight of caterpillars considerably increased when treated with Zn, Fe, and Zn/Fe (10, 20 mg; *p* < 0.001). In contrast, the LW larval weight of Zn-, Fe-, and Zn/Fe (40 mg)-exposed caterpillars significantly declined (*p* < 0.001) (Figure 3A). Compared to caterpillars treated with PQ, LW increased with 10 and 20 mg of Zn/PQ, Fe/PQ, and Zn/Fe/PQ exposure (*p* < 0.001). LW was lower following exposure to 40 mg Zn/PQ and Fe/PQ (*p* < 0.001). Interestingly, 40 mg Zn/Fe/PQ exposure mitigated compound toxicity, with a significant increase in LW (*p* < 0.001) (Figure 3B).

After 48 h of pre- and post-PQ exposure, the pupal weight (PW) changed (*p* < 0.001, F = 125.6, DF = 6; *p* < 0.001, F = 136, DF = 6). The Zn, Fe, and Zn/Fe treatments were considerably reduced PW overall. However, the Zn/Fe treatment distinctly declined for all applied doses (*p* < 0.001) (Figure 4A). Compared to PQ-treated larvae, PW increased in Zn/PQ, Fe/PQ, and Zn/Fe/PQ treatments at 10 mg exposure (*p* < 0.001). Of note, 40 mg exposure resulted in zero pupal ecdysis due to the 100% mortality of larvae (Figure 4B).

### 3.3. Effect of Single and Mixed Interactions of Zn, Fe, and PQ on Diet Consumption and Frass Production

**Figure 5:**
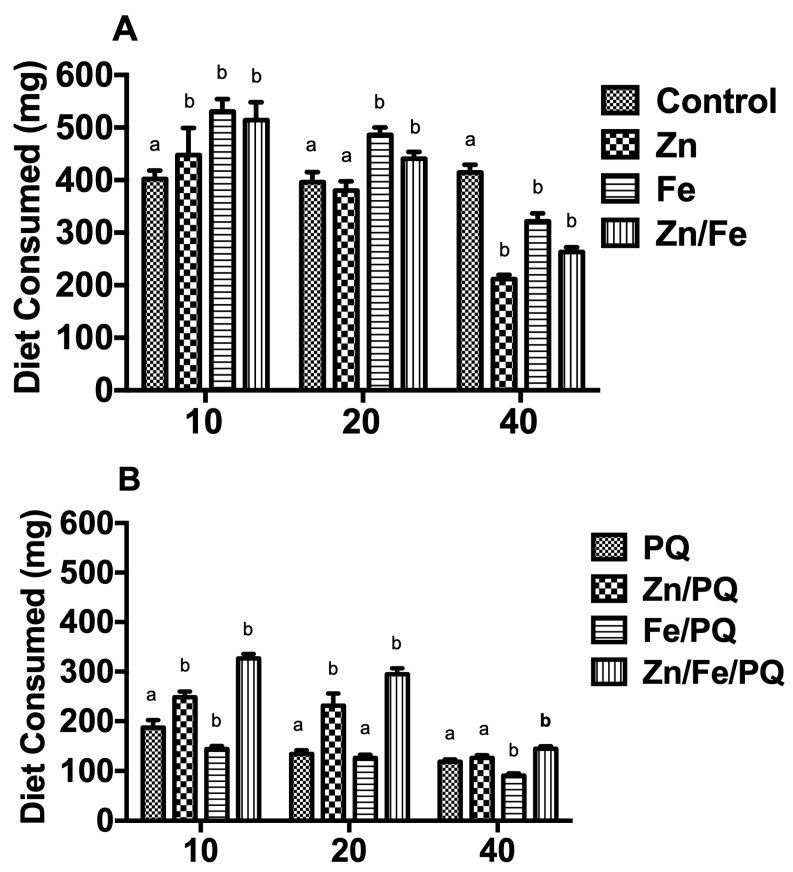
(**A**,**B**) Amounts of food consumed (mean values in mg per larvae) for each dose and compound treatment (4th instar until 48 h). Each treatment included 15 larvae, with 360 in total being tested. The experiment was repeated three times. Points on the bar graphs represent mean ± SEM. Different letters indicate significant differences in the treatment groups with the respective controls (pre-PQ: controls; post-PQ: PQ) (two-way ANOVA, Bonferroni post-tests, *p* < 0.05).

Single and mixed interactive effects were recorded for diet consumption after 48 h (Figure 5A). Compared to the control, pre–post-PQ food consumption increased to 10 mg and 20 mg for Zn, Fe, and Zn/Fe. In contrast, it decreased to 40 mg for Zn, Fe, and Zn/Fe (*p* < 0.001). Food consumption increased to 10 mg Zn/PQ and Zn/Fe/PQ, 20 mg Zn/PQ and Zn/Fe/PQ, and 40 mg Zn/Fe/PQ (*p* < 0.001). In contrast, decreases were recorded at 10 and 40 mg Fe/PQ (*p* < 0.001) (Figure 5B).

**Figure 6:**
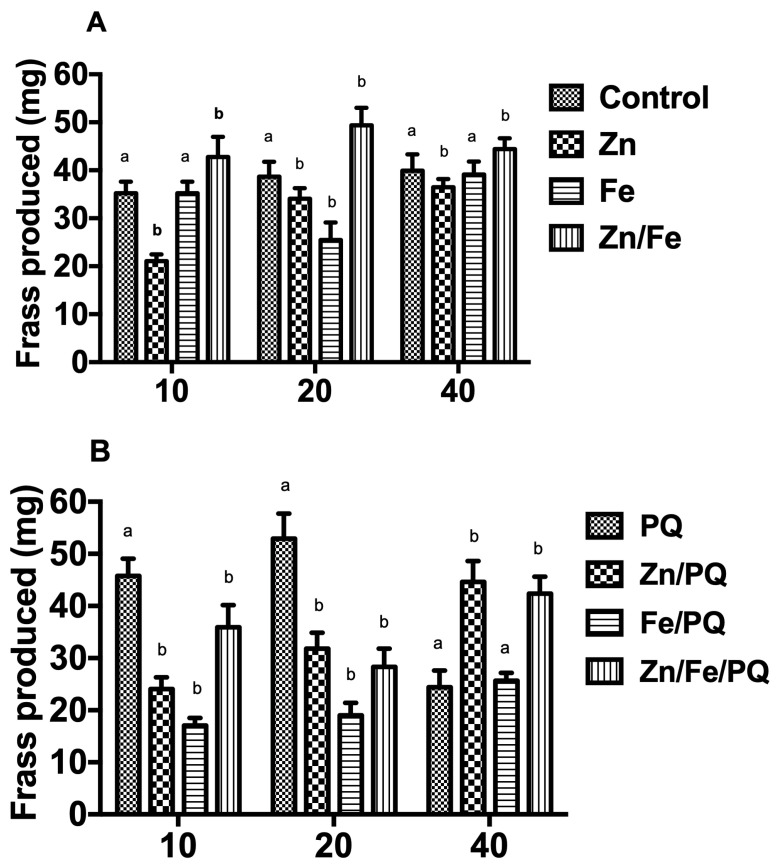
(**A**,**B**) Amount of frass excreted (mean values in mg per larvae) during the dose and compound treatments (4th larval instar until 48 h). Each treatment included 15 larvae, with 360 being tested in total. The experiment was repeated three times. Points on the bar graphs represent mean ± SEM. Different letters indicate significant differences in the treatment groups with the respective controls (pre-PQ: controls; post-PQ: PQ) (two-way ANOVA, Bonferroni post-tests, *p* < 0.05).

Frass production during pre-post PQ exposure varied with the interaction of doses and compounds (*p* < 0.0001, F = 76.3, DF = 6; *p* < 0.0001, F = 245, DF = 6). Compared to control larvae, frass production increased with Zn/Fe (*p* < 0.001) at all exposed doses. In contrast, it decreased to 10, 20, and 40 mg for Zn and 20 mg for Fe (*p* < 0.001) (Figure 6A). Frass production declined in Zn/PQ, Fe/PQ, and Zn/Fe/PQ at both 10 mg and 20 mg (*p* < 0.001). Co-exposure to Zn/PQ and Zn/Fe/PQ caused frass production to increase at 40 mg (*p* < 0.001) (Figure 6B).

### 3.4. Influence of Single and Mixed Interactions of Zn, Fe, and PQ on Pupal Death, Pupal and Imago Ecdysis, and Metamorphosis-Related Deformity

Pupal mortality, pupal and imago ecdysis, and overall deformities clearly showed the effects of single and combined exposures (Table 2). Fe exposure caused 15% (10 and 20 mg) and 29% (40 mg) larval death. The toxicity of single and combined metals with PQ was generally dose-dependent, with the lowest and highest larval mortality occurring at 10 mg and 40 mg exposure, respectively. PQ-exposed larvae had the highest mortality, with 29% (10 mg), 58% (20 mg), and 100% (40 mg) mortality. The interaction of PQ with Zn and Fe reduced mortality at low exposure, with 15% (10 mg) and 43% (20 mg) mortality. In contrast, 40 mg exposure had 100% mortality. Interestingly, the interaction of Zn/Fe/PQ rescued larval mortality, with 0% (10 mg), 29% (20 mg), and 72% (40 mg) mortality. The length of larval mortality varied greatly and was noticeably influenced by single and combined interactions and doses (Table 2).

Pupal ecdysis showed dose-dependent variation, with a minimal effect on pre-PQ exposure 100% (10 and 40 mg). Exposure to 20 mg Fe and Zn/Fe resulted in 94% and 89% larval ecdysis, respectively. PQ exposure reduced larval ecdysis; however, its interaction with metals restored ecdysis in a dose-dependent way. Zn/Fe/PQ interaction had the highest pupal ecdysis at 92% (10 mg) and 81% (20 mg). The length of pupal emergence was dose-dependent, with pre–post PQ exposure emergences of 6–10 days at 10 mg, 5–18 days at 20 mg, and 6–8 days at 40 mg. There was no pupal emergence with post-PQ exposure (Table 2).

Significant dosage and treatment differences were recorded for ecdysis to imago. Pre-PQ treatments had almost no toxic effects at 10 and 20 mg of Zn and Fe (100% ecdysis). In comparison, 40 mg had the lowest (42%) emergence for Fe, Zn (59%), and Zn/Fe (65%). PQ had the maximum toxicity on imago emergence, with 42% (10 mg) and 22% (20 mg) emergence. Combined exposure with Zn and Fe recovered ecdysis, while Zn/Fe/PQ had maximum ecdysis at 76% (10 mg) and 60% (20 mg) (Table 2).

**Figure 7:**
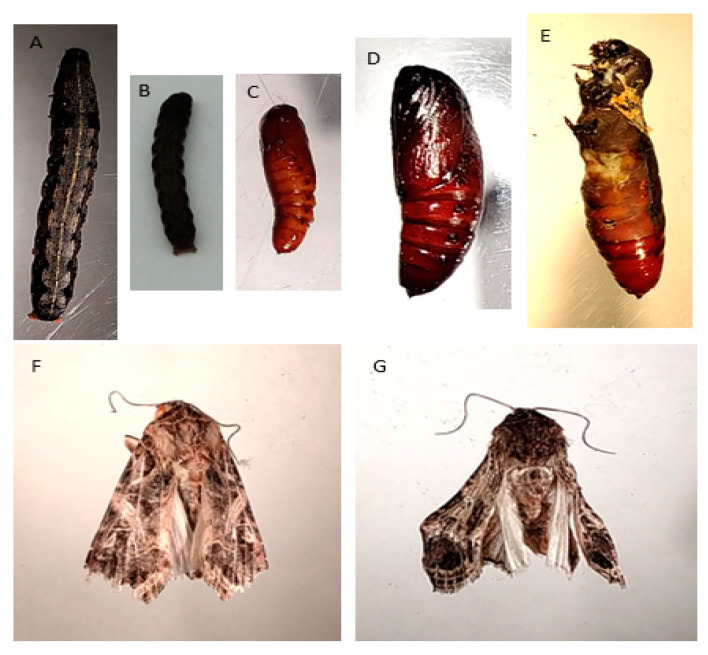
(**A**–**G**): Morphotoxicity of metals and herbicide co-exposure on *S. littoralis*. (**A**): Control larvae; (**B**) highly desiccated larvae that lost water after metal–herbicide treatment; (**C**) small pupae treated after post-PQ treatment; (**D**) control pupae; (**E**) larval-like body shape combined with pupal-like cuticle: metal–herbicide exposure; (**F**) control Adult; (**G**) wing defects caused by ZP (Zn/PQ) and FP (Fe/PQ) treatment.

Prominent defective moult blocks were obsreved at different stages of the larval and pupal moulting process. Regionally restricted moulting, moults changing to “intermediates” by combining the regions of newly secreted larva and pupal cuticles, and wing deformities in recently emerged imagoes were also observed, and they were recorded as the outcome of the single and mixed interactions of metals and PQ (Figure 7).

### 3.5. Antibiotic Effect: Influence of Zn and Fe Interactions over PQ on Haemolymph Bacteria

**Figure 8:**
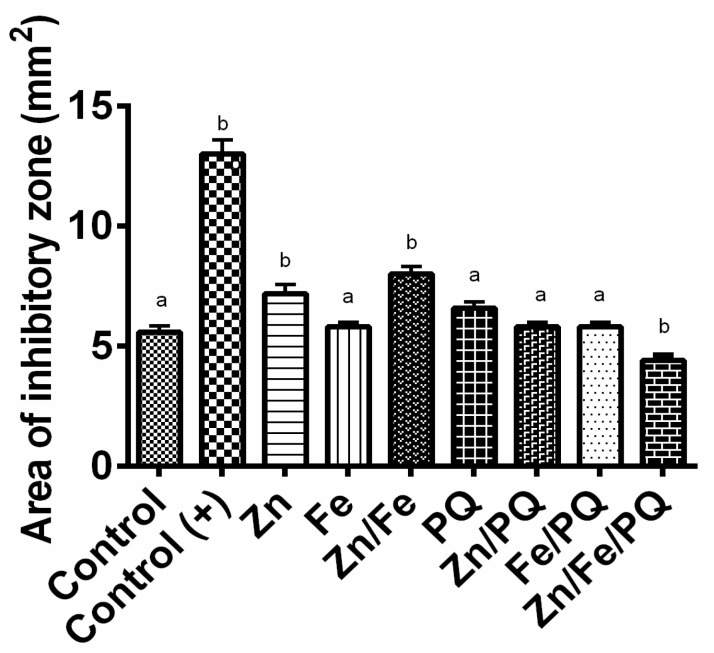
Effect of single and co-interactions of metals (ZnSO_4_ and FeSO_4_) and PQ on the gut haemocoel bacteria of *Spodoptera littoralis* along with different metal treatments, including paraquat and the positive control streptomycin after 3 days incubation on NA (nutrient agar) plates. The bar graphs are representative of 30 μg of either metals or the combination with PQ or streptomycin on a paper disc of size 0.5 mm placed on NA plates. Each treatment included 5 larvae, and in total, 40 were tested. The experiment groups with the prospective controls (pre-PQ: controls; post-PQ-PQ) (one-way ANOVA, Dunnett’s multiple comparisons tests, *p* < 0.05; DF = 6) were repeated thrice. Points in the bar graphs represent mean ± SEM. Different letters indicate significant differences in the treatment.

The possible antibiotic effect of metals, herbicide, and their combination on hemolymph bacteria with the disc susceptibility assay exhibited a high inhibition zone around the disc with Zn (*p* < 0.001) and Zn/Fe (*p* < 0.001), showing that streptomycin inhibited the growth of hemolymph bacteria. In contrast, Fe alone showed no inhibitory effect when compared to the control. Zn/PQ, Fe/PQ, and Zn/Fe/PQ showed reduced inhibition towards hemolymph bacteria. At the same time, the highest reduction showing a difference with the control occurred with Zn/Fe/PQ (*p* < 0.01) (Figure 8).

## 4. Discussion

This study showed that higher doses (40 mg) noticeably lowered *Spodoptera littoralis* larvae survival, and this was dependent on the interaction of exposed agents. Lower doses (10 and 20 mg) impacted survival, with specificity to the chemicals and their combinations (Figure 2). Of note is the co-exposure of Zn/Fe, which enhanced larval survival compared to single exposure (100% at 10 and 20 mg; 85% at 40 mg). Unexpectedly, higher doses of Zn/Fe/PQ reversed harmful effects and promoted survival. It is possible that the interaction of these agents alters their specificity or causes the chelation of metals, reducing PQ-induced oxidative stress and toxicity. For instance, Erre et al.. [31] showed that the herbicide “Imuznpyr” provides efficient chelating abilities and interacts strongly with cobalt (Co), manganese (Mn), and nickel (Ni) ions via a mono-negative anionic ligand. Thus, it is important to elucidate such interactions given the impacts of both metal and herbicide pollutants on soil quality and insect population dynamics. Low concentrations of Zn, such as 112 μg per 100 mL diet, have been shown to promote growth in aphids [32]. Our findings align with these earlier studies, demonstrating an increase in larval weight under both single and combined doses of Zn, Fe, and Zn/Fe (10 and 20 mg). Conversely, exposure to high concentrations of Zn, Fe, and Zn/Fe (40 mg) adversely affected growth and development. Similar findings have been reported in previous studies, where the effective co-stress concentrations of Cd and Zn were 40 mg/kg and 400 mg/kg, respectively [33].

Jin et al. [5] also showed that high concentrations of Zn (750 mg/kg) significantly inhibited the development and weight gain of *S. litura*. Thus, surviving *S. littoralis* might expend more energy from food on Zn detoxification versus development [34]. Data on the lethal concentrations of LC50 Zn toxicity to *S. littoralis* exist [23], along with tests at very high concentrations (150–750 mg/kg) to determine detrimental effects [5,35]. Likewise, laboratory studies showed that excess Fe affects its metabolism and enhances insect mortality through oxidative stress [27,36].

In our study, PQ alone clearly inhibited the growth of *S. littoralis* larvae, yet its co-exposure to Zn and Fe considerably recovered growth. Thus, PQ likely interacts with the two metal ions to form complexes that mitigate toxicity. Further studies on this need to be carried out. For instance, Erre et al. [31] provided evidence of herbicide metal complexes with efficient chelating abilities using single-crystal X-ray diffraction analyses. Furthermore, Chang and Kao [29] showed that metal chelators (Fe and Cu) effectively reduce PQ-mediated toxicity. In our study, Zn, Fe, and Zn/Fe exposure tended to decrease PW in comparison to the control. Sell and Schmidt [36] showed that incorporating high doses (500 mg/kg) of chelated Cu, Fe, and Zn into the diet prevented pupae formation in *Trichoplusia ni* Hübner (Lepidoptera: Noctuidae). In our study, PQ exposure considerably reduced PW, whereas exposure of larvae to PQ with Zn and/or Fe (10 mg) significantly increased PW. The toxicity of PQ was likely limited through reduced oxidative stress and elevated superoxide dismutase and glutathione reductase activity [37]. In our study, larvae pupated were exposed to lower doses of PQ (10 and 20 mg) combined with metals. Thus, larvae are likely able to store or detoxify these substances in various regions of the body. However, pupation did not occur at the higher dose (40 mg). Ballan-Dufrançais [38] also reported that high doses of metals interfere with the enzymes required for certain hormone processes responsible for insect metamorphosis [39,40], preventing pupal formation.

In our study, pre-PQ diet consumption was higher than that of post-PQ; thus, PQ inclusion in the diet appeared to cause stress and a deterring effect in larvae, reducing their capacity to eat. Higher doses of PQ (40 mg) significantly decreased food consumption compared to lower doses (10 and 20 mg), likely because of stress and deterrent factors [41] (Figure 5A,B). Larval death was considerably lower when larvae were treated with Zn/Fe/PQ compared to PQ alone or co-exposure with Zn or Fe (Figure 2 and Table 1). Our findings support those of [42], who showed that melatonin (Mel) improved locomotor activity and longevity in transgenic knockdown Parkinson *D. melanogaster* exposed to PQ or PQ/Fe.

Metals and herbicides are endocrine-disrupting substances. In our study, the malformation of larvae and pupae was recorded in both pre- and post-PQ treatments; thus, higher and lower concentrations of metals and herbicides alone cause hormonal imbalance. Any change to Zn homeostasis potentially disrupts the proper morphogenesis and growth of organisms [43]. Herbicide and metal complexes appear to deter food intake, leading to starvation and desiccated larvae and small sized pupae or adults. Furthermore, some larvae that pupate might have abnormalities, with larval-like bodies combined with a pupal cuticle [44]. This phenomenon was documented at different concentrations of metals/herbicides in our experiments, regardless of being pre- or post-PQ treatment. At higher concentrations, especially for PQ combined with metals, wing defects were recorded in Zn/PQ and Fe/PQ, with short survival periods in adults (Figure 7). Sridhara and Bhat [45] and Sell and Schmidt [36] also recorded developmental abnormalities and the inhibition of pupation in the cabbage looper *T. ni* when larvae were fed a diet mixed with 500 mg/kg Chelatex Zn. This research revealed that co-exposure to zinc and iron reduces paraquat herbicide toxicity at sublethal levels, which may be viewed as a significant turning point in agroecosystems and pest management.

The disc diffusion assay revealed that Zn and Fe in combination with PQ influence the capacity of insects to respond to their immune stimulation, thereby regulating the metabolic and physiological parameters of *S. littoralis*. This is because metals and herbicides directly or indirectly influence hemolymph bacteria in the insect body through the production of antimicrobials or antibiotics. Nevertheless, the intake or uptake of metals, herbicides, and metal–herbicide complexes depend on their regulators. It has been stated that Fur, an iron uptake regulator present in the hemolymph, plays a key role in the defence against ROS damage and peroxide stress, whereas the Zn regulator (Zur) exhibits reduced virulence within numerous animals and plant models of microbial infection [46]. Further elaborate studies on this issue may improve our understanding of the metabolic functions of hemolymph microorganisms [47,48]. In our experiment, the differences between low and high concentrations of the tested agents had no significant differences on hemolymph bacteria. We recorded the order of toxicity towards hemolymph bacteria as Zn/Fe > Zn > PQ > Fe > Zn/Fe/PQ, indicating that interactive combinations might influence metabolism-promoting immunity and partial resistance. 

## 5. Conclusions

The toxicity of PQ to *Spodoptera littoralis* larvae was significantly reduced when combined with Zn and Fe. Remarkably, the synergistic “cocktail effect” of these pollutants mitigated the overall toxicity compared to their separate exposures. This phenomenon can be attributed to the formation of metal complexes with potent chelating properties, effectively reducing oxidative stress. Lower sublethal doses of the metal–herbicide combination further enhanced larval survival by stimulating critical physiological processes, such as increased diet consumption, frass production, and successful larval-to-pupal ecdysis.

Additionally, the Zn/Fe/PQ interaction exhibited a notable immunomodulatory effect on haemolymph, indicating a regulatory influence on gut microbial dynamics. These findings underscore the complex interplay between pollutants and their physiological impacts on pest species. Our study has significant implications for understanding physiological toxicity mechanisms and developing innovative pest management strategies in agricultural ecosystems.

## Figures and Tables

**Table 1:** Lethal concentration (LC20) values of tested compounds.

Compound	LC20(mg/kg Diet)	Fiducial Limits		Slope
Metals and Herbicide		Lower	Upper	
Zinc (Zn)	23.757	6.81	33.714	5.474 ± 1.235
Iron (Fe)	23.958	14.783	30.731	4.990 ± 1.091
Paraquat (PQ)	17.979	4.054	28.17	4.108 ± 0.832

**Table 2:** Summary of single and co-exposure of Zn, Fe, and PQ on pupal death (% days) and pupal and imago ecdysis (% days) of *S. littoralis* (*N* = 360 larvae in total; 15 larvae per test). The experiments were completed in triplicate, with the results presenting the combined data. Note: ^a^ Pupal malformation. Days are counted from the day of the indicated treatment (start of the penultimate larval instar) until imago ecdysis.

Treatment	Pupal Ecdysis (%)/Days			Pupal Death (%)/Days			Ecdysis to Imago (%)/Days		
	10 mg	20 mg	40 mg	10 mg	20 mg	40 mg	10 mg	20 mg	40 mg
Control	100/7–9	100/6–7	100/6–8	0	0	0	100/5–8	100/4–6	100/6–7
Zn	100/6–9	100/6–7	100/7–8	0	0	0	100/6–9	100/5–7	59/8–9
Fe	100/6–8	94/7–8	100/7–8	0	6/6 ^a^	0	100/7–9	100/3–7	42/7–9
Zn/Fe	100/6–8	89/5–9	100/7–8	0	11/5 ^a^	0	100/9–10	88/7–10	65/10–12
							42/10		0
PQ	76/8–10	50/5–9	0	10/6 ^a^	23/7 ^a^	100		22/8–12	
							57/10–12		0
Zn/PQ	81/8–9	64/7–10	0	12/8 ^a^	15/6–7 ^a^	100	61/10–12	35/7–12	
							76/10		0
Fe/PQ	67/8–9	49/6–11	0	11/7 ^a^	15/8–9 ^a^	100		39/7–12	
									0
Zn_/_Fe_/_PQ	92/9–6	81/6–18	0	5/12	7/9–10	100		60/10–12	

## Data Availability

Data will be made available by the corresponding author upon request.
